# Dengue: Update on Clinically Relevant Therapeutic Strategies and Vaccines

**DOI:** 10.1007/s40506-023-00263-w

**Published:** 2023-04-18

**Authors:** Monica Palanichamy Kala, Ashley L. St. John, Abhay P. S. Rathore

**Affiliations:** 1 Program in Emerging Infectious Diseases, Duke-National University of Singapore Medical School, 8 College Rd., Level 9, Singapore, 169857 Singapore; 2 Department of Microbiology and Immunology, Yong Loo Lin School of Medicine, National University of Singapore, Singapore, Singapore; 3 SingHealth Duke-NUS Global Health Institute, Singapore, Singapore; 4 Department of Pathology, Duke University Medical Center, 207 Research Rd, Durham, NC 27705 USA

**Keywords:** Dengue fever, Vascular leakage, Therapeutics, Vaccines, Metabolic disorders, Mast cells

## Abstract

Dengue viruses (DENV) continue to circulate worldwide, resulting in a significant burden on human health. There are four antigenically distinct serotypes of DENV, an infection of which could result in a potentially life-threatening disease. Current treatment options are limited and rely on supportive care. Although one dengue vaccine is approved for dengue-immune individuals and has modest efficacy, there is still a need for therapeutics and vaccines that can reduce dengue morbidities and lower the infection burden. There have been recent advances in the development of promising drugs for the treatment of dengue. These include direct antivirals that can reduce virus replication as well as host-targeted drugs for reducing inflammation and/or vascular pathologies. There are also new vaccine candidates that are being evaluated for their safety and efficacy in preventing dengue disease. This review highlights nuances in the current standard-of-care treatment of dengue. We also discuss emerging treatment options, therapeutic drugs, and vaccines that are currently being pursued at various stages of preclinical and clinical development.

## Introduction

Dengue fever (DF), caused by the dengue virus (DENV), is the world’s most prevalent and important arboviral infection. It is estimated that nearly 390 million infections occur annually, of which 96 million manifest clinically [1]. DENV belongs to the genus Flavivirus, which also comprises several other clinically important human pathogens, such as Zika, Japanese encephalitis, West Nile, and Yellow Fever viruses, among others. The DENV genome consists of a single-stranded positive-sense RNA that encodes for three structural (capsid, prM/M, and E) and seven non-structural proteins that are translated during the virus replication cycle [2]. The virus is spread by an infected mosquito bite during a blood meal and exists in both sylvatic and urban ecosystems [3]. The sylvatic cycle of DENV involves virus transmission between non-human primates (NHP) and mosquitos prevalent in the forest [4, 5] while in the urban cycle of transmission, the virus is maintained within human population aided by urban dwelling mosquitos such as *Aedes aegypti* [6]. As the geographical distributions of these vectors are expanding it is likely that DENV will spread further [7]. There are 4 serotypes of DENV (DENV1-4), which makes it likely that an individual will be exposed to the virus multiple times in their lifetime [8]. Latest models of dengue transmission estimate that 4 million cases require hospitalization each year [9] and account for an annual estimated cost of US $8.9 billion globally [10]. With an increase in urbanization and climate change supporting the spread of the mosquito vector, some models predict an increasing risk of DENV transmission, potentially impacting 6.1 billion people by 2080 [11].

## Dengue clinical course

**Fig. 1:**
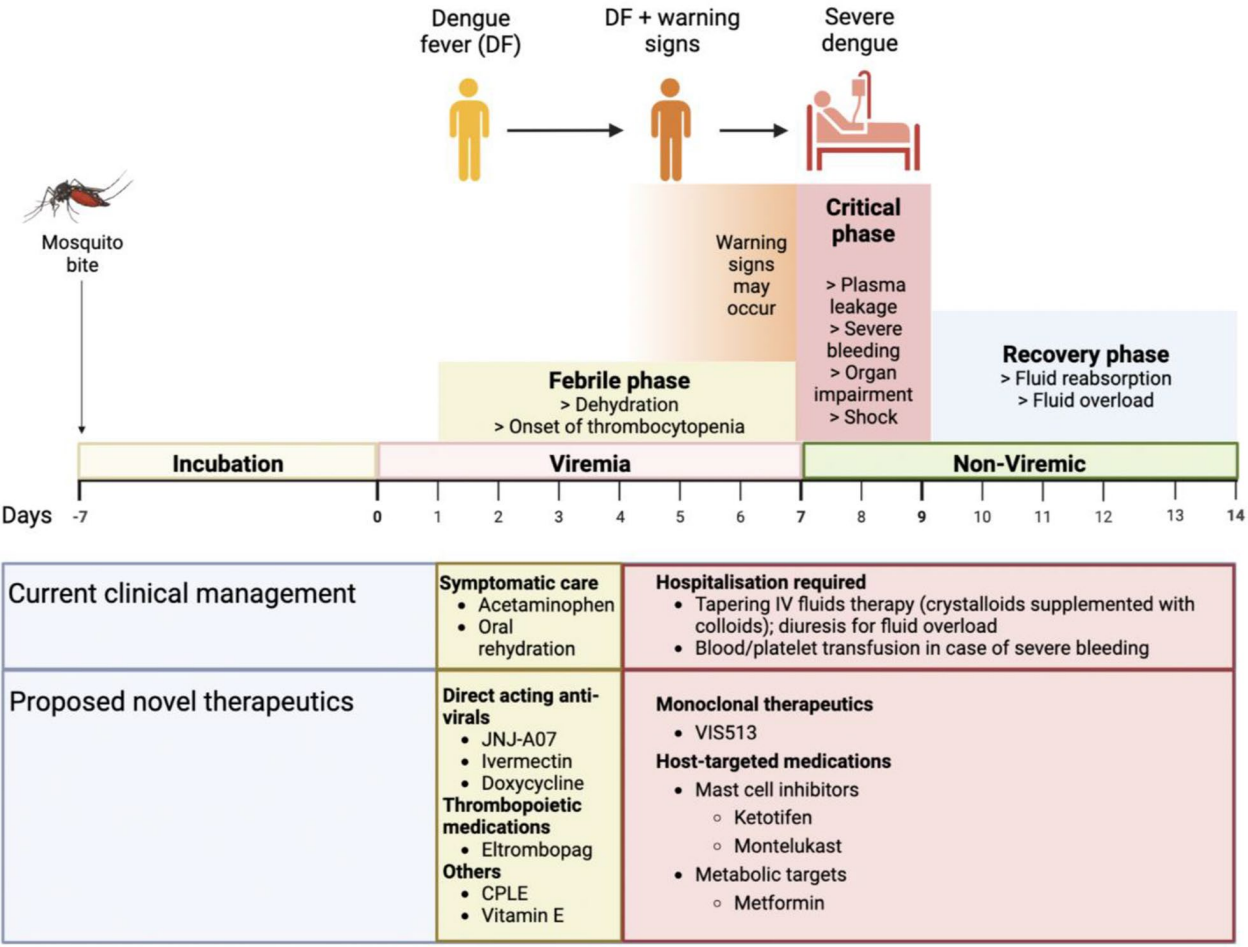
The clinical management and possible treatment options for dengue disease. Diagram was made using biorender.com

The clinical course of DF begins with common flu-like symptoms, including fever, nausea, myalgia, and headache [12]. Although DF is a self-limiting mild disease, some patients will develop the severe form of the disease, characterized by plasma leakage, hemorrhaging, and shock [12]. According to the 2009 WHO revised guidelines, dengue disease is categorized as DF, DF with warning signs, and severe dengue [13] (Fig. 1). Some of the warning signs include hepatomegaly, abdominal pain, mucosal bleeding, and increasing hematocrit concurrent with rapidly declining platelets [13]. In general, there are three phases of dengue disease—the febrile phase when viremia is high, the critical phase when fever and viremia are resolving, but the patient may experience thrombocytopenia and or plasma leakage that manifests as hemoconcentration and fluid accumulation in tissues in severe cases, and, finally, the convalescent phase with fluid reabsorption and recovery [13] (Fig. 1). During the critical phase, if vascular complications and hemorrhage occur, the disease may also be called dengue hemorrhagic fever (DHF). If left untreated, patients with DHF can develop multiorgan failure and shock, known as dengue shock syndrome (DSS) [13]. Case fatality rates (CFRs) vary among countries but can be as high as 10–15% in some and < 1% in others, depending mostly on access to and quality of healthcare [14, 15].

## Dengue risk factors

Secondary heterologous dengue infection is the most well-established risk factor for severe dengue. This is thought to be attributable to antibody-dependent enhancement of infection (ADE), which occurs when viruses bound to sub-neutralizing antibodies are opsonized by immune cells like dendritic cells, monocytes/macrophages through Fc receptor, which then results in increased virus production [8]. Moreover, cross-reactive non-neutralizing heterotypic antibodies can also lead to increased antibody dependent cellular cytotoxicity (ADCC) and excessive activation of mast cells, resulting in release of vasoactive mediators that have been shown to promote dengue vascular pathology [16, 17•, 18]. Other risk factors (reviewed elsewhere [19]) include host factors like pre-existing metabolic diseases, age, gender, and HLA type; and may also include viral factors like viral load, NS1 antigenemia. Healthcare quality and access also influence the risk for disease progression.

## Updates in dengue supportive care

In the absence of targeted dengue therapeutics, good supportive care, with treatment for symptom management and fluid administration, is the cornerstone of clinical management of dengue (Fig. 1). WHO guidelines recommend acetaminophen or paracetamol for antipyretics, with a recommended dose of 10 mg/kg/dose. The maximum dose for adults is 4 g/day and a frequency of not less than 6 h [13]. However, a recent double-blind, randomized, placebo controlled clinical trial (NCT02833584) raises concerns on the safety and efficacy of paracetamol in dengue patients [20•]. The study found that compared to placebo, administering 500 mg of paracetamol every 4 h (median dose was 1.5 g/day) when body temperature exceeded 38 °C had significantly higher rate of transaminase elevation (22% vs 10% in placebo; incidence rate ratio 3·77, 95% CI 1·36 − 10·46, *p* = 0·011). Considering that hepatic dysfunction is a common complication of dengue and elevated liver transaminases are consistently associated with severe dengue [21–24], this trial raises concerns of hepatotoxicity of paracetamol, even at therapeutic doses, in a situation when the hepatocytes are already stressed [25]. Moreover, paracetamol intake did not alter mean and maximum body temperatures, duration of fever, length of hospitalization, analgesic intake, and mean and maximum pain score, compared to the placebo group, suggesting a lack of defervescent or analgesic benefit, although the study was under-powered for assessment of this secondary outcome. Since the current WHO guidelines contraindicate aspirin and other non-steroidal anti-inflammatory drugs (NSAIDs) due to their antiplatelet activity and bleeding risk and paracetamol is the only available therapeutic option, caution should be exercised when prescribing paracetamol and transaminase levels should be monitored.

Judicious fluid administration to ensure adequate tissue perfusion during the critical phase of illness is the other arm of mainstay dengue management. Oral rehydration with fluids other than plain water such as milk, fruit juice, oral rehydration solution (ORS), rice or barley water, and isotonic electrolyte solution is recommended in cases of mild dengue fever in patients with able oral intake [26, 27]. Hospitalization and intravenous (IV) fluid therapy are recommended in cases of insufficient oral intake, emesis, a continuous rise in hematocrit (HCT) of 10–20% despite oral rehydration, development of warning signs, and in case of impending shock/shock [26]. Randomized clinical trials (RCTs) have evaluated both crystalloids and colloids for pediatric patients with DSS [28–30] and found no difference between the treatment groups with regards to the need for rescue resuscitation with colloids or shock recurrence [28]. However, in the case of profound shock when patients have pulse pressure < 10 mm Hg, colloids are preferred since they have been shown to restore cardiac index and hematocrit more rapidly than crystalloids [28–30]. Correspondingly, current WHO guidelines recommend volume replacement with isotonic crystalloid solutions as 10 ml/kg over 1 h for patients with compensated shock and 20 ml/kg bolus over 15 min for profound decompensated, hypovolemic shock, followed by a tapering fluid regimen supplemented with bolus colloid solutions as necessary [26, 27]. However, there have been limited clinical trials with evidence to support the current WHO fluid resuscitation regimen recommendations for patients during the critical phase of the illness. This is concerning, particularly in the evidence of observational studies reporting longer duration of IV fluid therapy, greater amounts of IV fluid therapy, and IV fluid bolus as independent risk factors for respiratory distress with fluid accumulation [31], a potential iatrogenic complication of dengue supportive treatment. Therein, fluid regimens that taper and restrict fluids as needed during the recovery phase of dengue are necessary to prevent fluid overload complications (Fig. 1). Currently, several RCTs have been registered to evaluate WHO-recommended fluid regimens for dengue patients with warning signs and for patients in the early stages of DHF: CTRI/2019/09/021026 aims to compare need-based vs guidelines-based fluid administration in dengue patients with warning signs; CTRI/2020/01/022694 aims to evaluate respiratory distress in children with dengue warning signs receiving current WHO-recommended fluid regimen vs an alternate tapering fluid regimen. Administration of albumin in crystalloid refractory shock was associated with shock resolution with reduced fluid overload symptoms in an observational study [32]. RCTs (CTRI/2018/03/012781 and NCT04076254) have been registered to evaluate the efficacy of albumin administration in addition to normal (0.9%) saline for effective fluid resuscitation of severe dengue patients. A multi-center double-blind parallel group RCT has also been registered (SLCTR/2022/003) in Sri Lanka to evaluate the effectiveness of Dextran 40 compared to 0.9% saline in preventing dengue shock in the early leakage phase of DHF. Results from these on-going trials will be important to identify the most effective fluid therapy with a reduced risk of iatrogenic complications for dengue case management. Additional considerations for fluid replacement have been reviewed elsewhere [33].

In the context of hypotensive shock without a rise in hematocrit, significant internal bleeding should be suspected. Current guidelines are to transfuse aliquots of 10 ml/kg of whole blood or 5 ml/kg of packed red blood cells and monitor the clinical response and posttransfusion hematocrit [26, 27]. Thrombocytopenia (platelet count below 100 × 10^9^/L) is a hallmark of dengue infection and has been shown to have various etiologies including enhanced platelet clearance [34•, 35]. Low platelet levels are associated with severity of dengue disease [34•, 35–37] since thrombocytopenia can precipitate hemorrhage. As per current guidelines, strict bed rest, and avoidance of NSAIDs or any source of trauma have been the only courses of action advised for patients with severe thrombocytopenia (platelet count below 20 × 10^9^/L) since a RCT (NCT01030211) found that prophylactic platelet transfusion was not superior to supportive care in preventing bleeding and, in fact, increased adverse reactions [26, 38]. As such, effective drugs to treat thrombocytopenia are needed. A recent phase II open-labeled RCT evaluating the efficacy of eltrombopag to correct thrombocytopenia in moderate to severe dengue patients with platelet counts below 100 × 10^9^/L (SLCTR/2019/037) shows potential [39•]. Administration of 25 mg of eltrombopag, a thrombopoietin receptor agonist that stimulates megakaryopoiesis [40], in a short regimen for three days, was shown to significantly augment platelet recovery and increase platelet count to above the lower normal limit (LNL) (150 × 10^9^/L) in 91% of patients on day-7 post enrollment, compared to 55% in the control group. Moreover, eltrombopag abated bleeding manifestations in 93% of grade II DHF patients by day-7, while intermittent bleeding with low platelet counts was still observed in 40% of patients in the control group. Eltrombopag also had a favorable safety profile with no thrombosis and no increase in adverse events (vomiting, diarrhea) compared to the control group. These results suggest that eltrombopag may be a therapeutic option for thrombocytopenia and for abating bleeding manifestations in dengue patients.

It is to be noted that previous interventional [38, 41] and retrospective studies [42] have shown that increasing platelet counts via transfusion neither prevented the development of severe bleeding nor shortened time to cessation of bleeding. Analysis of patient samples and supporting in vitro studies suggest that this could be due to DENV infection and the subsequent activation and consumption of platelets, resulting in increased thrombus formation [43]. Therefore, increasing platelet count may not necessarily contribute to bleeding resolution and could result in increased intravascular coagulation since platelet activating factors may still be present in the circulation [44]. More trials are needed to understand the clinical efficacy of adding platelets, including through mechanisms to increase platelet production. Specifically the rate of prevention of severe bleeding, rate of bleeding resolution, and normalization of other coagulation abnormalities reported during DHF [44] should be evaluated.

Desialylation or removal of sialic acid on platelet membrane by endogenous neuraminidase, induced by binding of von Willebrand factor (VWN) to platelets, has been suggested as possible mechanism for increased platelet clearance in dengue patients [45]. Based on this, oseltamivir, a neuraminidase inhibitor that is commonly administered for influenza [45] was tested as a potential therapeutic for thrombocytopenia in a phase 2, TOTO trial (Treatment Of Thrombocytopenia with Oseltamivir in acute dengue virus infection: a randomized, placebo controlled, multicenter trial) conducted in Indonesia (ISRCTN35227717) [46]. Unfortunately, the drug was found to be ineffective for both platelet recovery and preventing plasma leakage in patients with acute dengue with moderate to severe thrombocytopenia. This indicates that while many drugs may show potential as dengue therapeutics in preclinical development, proof of concept testing with clinical trials with predefined primary and secondary clinical outcomes is important.

## Development of therapeutics targeting dengue virus

Considering that a higher viral burden could promote severe dengue disease [47, 48], identification of DENV-specific antivirals has been an important focus of research for dengue therapeutics. So far, clinical trials with repurposed drugs with antiviral activity in pre-clinical studies like balapiravir [49], chloroquine [50], lovastatin [51], and celgosivir [52] did not show any efficacy in reducing viremia nor beneficial clinical outcomes. In this section, we describe both direct-acting and host factors-targeting antivirals that show the most potential as dengue therapeutics and are in advanced stages of clinical testing. These have also been summarized in Table 1 and Fig. 1.

**Table 1:** Summary of dengue therapeutics in advanced stages of clinical testing

Drug name	Target	Pre-clinical data	Clinical data
Treatment options with possible benefits			
JNJ-64281802	NS4B inhibitor that inhibits viral replication	Antiviral activity in vitro was shown for its analog, JNJ-A07. Decrease in viremia, viral burden, and inflammatory cytokines, and improved survival in immunocompromised mouse model of DENV infection [53•]	Clinical trials for dengue prophylaxis in healthy individuals (NCT05201794) as well as for dengue therapy in patients with confirmed dengue fever (NCT04906980) are in progress
Ivermectin	Antiviral agent that inhibits host nuclear import receptors	Reduced viral replication in vitro [56, 57]	Randomized double-blind placebo-controlled trial (*n* = 203) showed faster NS1 antigenemia clearance but no improvement in virological or clinical efficacy (NCT02045069) [58•]
AT-752	Guanosine nucleotide analog inhibiting NS5 RdRp function, inhibits viral replication	Reduced viremia on certain days and improved survival in immunocompromised mice models infected with DENV [63]	Phase 1 (NCT05366439) and phase 2 (NCT05466240) clinical trials are in progress
Doxycycline	Tetracycline class antibiotic, inhibits viral entry and replication by inhibiting NS2B-NS3	Antiviral activity against all four DENV serotypes in vitro [64]	One randomized clinical trial (*n* = 231) showed reduction in inflammatory cytokines and another case control study (*n* = 120) showed faster platelet recovery, leukocyte count, and reduced length of hospitalization. Clinical trials to test efficacy of doxycycline as a dengue therapeutic in pediatric (CTRI/2018/01/011548) and adult (CTRI/2021/09/036661) populations are on-going
Eltrombopag	Thrombopoietin receptor agonist	Nil	Randomized open-label placebo-controlled trial (*n* = 101) showed improved platelet recovery, increased platelet count, and reduced bleeding manifestations in grade II DHF patients (SLCTR/2019/037) [39•]
UV-4B	Endogenous alpha glucosidase inhibitor	Antiviral activity in vitro [70] and in vivo [71]	Phase 1a clinical trial (NCT02061358) with healthy subjects indicated that a single dose up to 1000 mg of UV-4B was safe and well tolerated [72]
Zanamivir	Neuraminidase inhibitor to block desialylation on platelet membrane	Reduction in DENV2 NS1-induced endothelial hyperpermeability and vascular leakage in vitro [73]	Clinical trial to test efficacy against vascular leakage (NCT04597437) is currently on-going
VIS513	Pan-serotype anti-DENV monoclonal antibody	Diminished circulating infectious DENV in NHPs [88], and reduced viral load with improved survival in immunocompromised mice models of DENV infection [87]	Clinical trial in progress (CTRI/2021/07/035290)
Ketotifen	Prevent mast cell degranulation and release of vasoactive products	Reduced vascular leakage in mouse models of DENV infection [16, 18]	Clinical trial in progress (NCT02673840)
Montelukast	Cysteinyl leukotriene receptor antagonist	Reduced vascular leakage in mouse models of DENV infection [18]	One randomized open-label clinical trial (*n* = 200) reported reduced incidence and relative risk of DSS (narrow pulse pressure < 20 mmHg and hypotension for age) [95]. A randomized, double-blind, placebo controlled, superiority trial (NCT04673422) to test efficacy of montelukast is currently on-going
Rupatadine	Dual PAF and histamine-1-receptor antagonist	Reduced vascular leakage in mouse model of DENV infection [94]	Randomized placebo-controlled trial (*n* = 183) did not show reduction in leakage, but improved platelet counts and liver enzyme values (SLCTR/2014/023) [94]
Metformin	Oral anti-hyperglycemic agent, AMPK activator	Antiviral effect in DENV infected cells in vitro [101]	A retrospective study (*n* = 223) showed decreased risk of severe dengue with metformin use in dengue patients with diabetes [103•]. Clinical trial in progress (NCT04377451) [104]
Carica papaya leaf extract	Anti-inflammatory agent, augments megakaryocyte development and platelet formation	Reduced production of proinflammatory cytokines [118] and increased platelet count in vivo [119]	Three randomized controlled trials showed an improvement in platelet counts [106–108]
Vitamin E	Unclear mechanism. Can act as an antioxidant	Nil	Randomized placebo-controlled trials showed faster platelet recovery (*n* = 66) [114] and reduced liver enzyme derangement (*n* = 127) (SLCTR/2015/012) [113]
Vitamin D	Unclear mechanism. Can increase calcium availability for immune cell activation	Reduced viral replication and inflammatory cytokines production in vitro [120]	Randomized clinical trial (*n* = 124) showed reduced relative risk for DHF (*P,* significance for trend was 0.0588) [115]
Vitamin C	Unclear mechanism. Can act as an antioxidant	Nil	Retrospective study (*n* = 200) found improved platelet recovery and reduced hospitalization duration in treatment group [117]. Clinical trials for Vitamin C alone (SLCTR/2017/028) and in combination with Vitamin B1 (CTRI/2019/09/021244) are in progress
Zinc	Unclear mechanism	Nil	Randomized double-blind placebo-controlled trial (*n* = 50) (TCTR20151110001) showed reduced hospitalization duration, but no improvement in other clinical or laboratorical outcomes [116]
Treatment options without support for benefits			
Paracetamol	Anti-pyretic	Nil	Randomized double-blind placebo-controlled trial (*n* = 123) showed worse liver enzyme derangements in the treatment group, with no improvement in other clinical or laboratorical parameters. (NCT02833584) [20•]
Oseltamivir	Neuraminidase inhibitor to block desialylation on platelet membrane	One ex vivo study showed that oseltamivir reduced desialylation on platelets from healthy donors [45]	Randomized double-blind placebo-controlled trial (*n* = 70) did not show improvement in platelet recovery or plasma leakage parameters (ISRCTN35227717) [46]

A direct-acting dengue therapeutic with promising preclinical data is the JNJ-A07, NS4B inhibitor developed by Janssen Pharmaceuticals [53•]. The drug was identified from a set of 2000 related molecules that were generated by modifying the structure of a ketoindole compound that showed inhibition against DENV2 infection [54]. The drug inhibited DENV replication complex formation by inhibiting the interaction between the NS4B protein, a multi-transmembrane protein at the endoplasmic reticulum, and NS3, a serine protease-helicase [55]. JNJ-A07 showed antiviral activity against all four dengue serotypes as well as 21 clinical isolates in vitro. Immunocompromised murine models infected with lethal as well as sub-lethal DENV2 doses and in models of antibody-dependent enhancement showed rapid decrease in viremia and viral burden in organs, regardless of whether JNJ-A07 treatment was started at the onset of infection or a delayed treatment. Reduction in pro-inflammatory cytokines like IL-18, IFN-γ, TNF, and IL-6 and increase in survival percentage were also observed in the drug-treated infected mice. Hemoconcentration or fluid accumulation in tissues and immune response parameters such as neutralizing antibody titers following infection in the drug-treated group were not assessed. This is important since the rapid suppression of viremia by the drug could lead to weakened antibody production that could precipitate severe dengue in a subsequent dengue infection. Currently, JNJ-64281802, an analog of JNJ-A07, has been registered for two phase 2, randomized, double-blind, placebo-controlled clinical trials to study its efficacy for dengue prophylaxis in healthy individuals (NCT05201794) as well as for dengue therapeutics in patients with confirmed dengue fever (NCT04906980).

Ivermectin, a broad-spectrum antiparasitic drug against helminth infection, has been previously shown to inhibit all four dengue serotypes in vitro by inhibiting the host nuclear import proteins that were important for nuclear localization of the dengue NS5 protein with RNA-dependent RNA polymerase (RdRp) function [56, 57]. A phase 2/3 randomized, double-blind, placebo-controlled trial (NCT02045069) was conducted to study the efficacy of a once-daily dose of ivermectin 400 μg/kg for 2–3 days in adult dengue patients [58•]. Interestingly, the study reported faster NS1 antigenemia clearance upon ivermectin treatment, with no difference in viremia, viral clearance, or any beneficial clinical outcomes including fever, DHF incidence, hospitalization, pleural effusion, hemoconcentration, or fluid requirements [58•]. High NS1 levels have been previously shown to be a risk factor for the development of dengue hemorrhagic fever [59] since it can induce pathologic complement activation [60] as well as independently induce vascular leakage [61] by disrupting the endothelial glycocalyx [62]. Hence, while this current trial did not report any clinical efficacy at this dosing regimen, further studies to understand the pharmacodynamics of ivermectin and its mechanism of action with regards to NS1 are warranted.

AT-752, an orally available guanosine nucleotide analog recently developed by Atea Pharmaceuticals, also functions by targeting the RdRp function of the NS5 protein [63]. The drug functions by metabolizing into an active triphosphate metabolite, AT-9010, intracellularly, which acts as a GTP analog and is incorporated into RNA by RdRp, therein inhibiting viral replication. Correspondingly, AT-281, the prodrug of AT-752 inhibited DENV2 and DENV3 in vitro and AT-752 showed reduced viremia (albeit only at day 6 and 8 post infection) and improved survival in DENV2-infected immunocompromised mice. Currently, the drugs are in phase 1 (NCT05366439) and phase 2 (NCT05466240) double-blind, randomized, placebo-controlled studies to assess the safety and antiviral activity in the dengue human challenge model and in dengue patients, respectively.

Doxycycline, a broad-spectrum tetracycline-class antibiotic and antimalarial, has shown some efficacy as an antiviral against DENV1-4 in vitro by inhibiting NS2B-NS3 protease activity, resulting in reduced viral entry and replication [64]. A randomized clinical trial (with no placebo control arm) in Brazil, testing doxycycline for its efficacy as an anti-inflammatory drug in dengue infection showed reduction in pro-inflammatory cytokines IL-6 and TNF in the treatment group compared to patients receiving standard symptomatic and supportive care [65]. A case–control study in India showed that doxycycline-treated dengue patients showed faster recovery of platelet and leukocyte counts and reduced hospital stay [66]. However, these studies so far have not been robust double-blind randomized placebo-controlled clinical trials with defined end point measurements. One such clinical trial (CTRI/2018/01/011548) is currently registered in India to study the efficacy of doxycycline as a dengue antiviral in a pediatric population and could clarify whether doxycycline has use as dengue therapeutic.

DENV infection and replication also require host factors that could be potential drug targets. N-linked glycosylation of viral proteins (pre-membrane (prM) and envelope (E) proteins) mediated by host α- glucosidase in the endoplasmic reticulum is important for viral assembly and subsequent release of mature, infectious DENV particles [67]. Celgosivir, an alpha-glucosidase inhibitor, had impaired folding and trapping of NS1 and significant antiviral activity when tested in vivo in mouse models of dengue infection [68]. Although the previous trial did not meet its primary outcome of reducing viremia or fever, subsequent evaluation of the pharmacokinetic endpoints of the trial inferred that a revised dosing regimen that would increase the steady-state trough concentrations of the drug in the patient serum might be more efficacious [52, 69]. UV-4B (N-(9′-methoxynonyl)-1-deoxynojirimycin), another alpha-glucosidase inhibitor, also showed antiviral activity against all four serotypes of dengue in vitro [70]. In vivo studies using immunosuppressed mice showed decreased viremia and viral burden and improved survival in a lethal ADE model of dengue disease [71]*.* A Phase 1a clinical trial (NCT02061358) with healthy subjects, evaluating the safety, tolerability, and pharmacokinetics of the drug, indicated that a single dose of up to 1000 mg UV-4B was safe and well tolerated [72]. The efficacy of this drug in dengue patients has yet to be determined.

Similar to oseltamivir mentioned earlier, Zanamivir, another neuraminidase inhibitor, has shown inhibition of DENV2 NS1-induced endothelial hyperpermeability in vitro by inhibiting endogenous sialidase [73]. Considering that the TOTO trial failed to show any clinical benefit, the efficacy of zanamivir in vivo remains speculative. A phase 1 pilot randomized, double-blind, placebo-controlled clinical trial for the evaluation of the safety and efficacy of five days of intravenous zanamivir treatment to treat vascular permeability syndrome (NCT04597437) is currently on-going.

There are several other direct-acting antivirals and host-directed antivirals (reviewed by others [74–77])—both novel and repurposed drugs, which are still in varying stages of pre-clinical development.

## Monoclonal therapeutics

An obstacle for the development of monoclonal antibody (mAb) therapeutics against DENV has been the identification of antibodies capable of cross-neutralizing DENV1-4, which make up a minority of antibodies induced by natural infection [78]. Many of the dominant antibodies following natural infection target the immunodominant epitopes of the E protein fusion loop and the prM proteins, which can be involved in ADE [79]. Most mAb development pipelines have focused on the goals of either developing broadly cross-neutralizing antibodies or producing antibody cocktails [80]. Cross-reactive neutralizing antibodies can be formed against domains I, II, and III of the E protein, yet those against the quaternary structure of E protein dimers are more likely to be potently cross-neutralizing [78, 79]. However, even highly potent neutralizing antibodies can induce ADE at intermediate concentrations [8, 81], emphasizing the importance of safety testing of mAbs to identify the optimal therapeutic window and concentration. The risk of ADE can also be mitigated by engineering LALA substitutions in the heavy chain [82, 83]. Monovalent mAb therapeutics also have the risk of becoming obsolete if viral immune evasion results in loss of neutralizing epitopes, as has been shown in the real world during the SARS-CoV-2 pandemic [84], and in animal models of DENV mAb testing [82]. Development of mAb as therapeutics or prophylactics for dengue has also been comprehensively reviewed elsewhere [85, 86] and three candidates for human use are currently in clinical trials.

VIS513, a humanized pan-serotype anti-DENV mAb developed by Visterra (Cambridge, Massachusetts) has been engineered to bind domain III of the E protein of all 4 DENV serotypes [87]. Administration of VIS513 either 24 h or 5 days post dengue infection in non-human primates was found to diminish infectious DENV in circulation without altering the endogenous antibody response [88]. In the murine model of antibody-enhanced DENV infection, VIS513 had increased protection of immunocompromised mice from lethal primary and secondary infection [87]. VIS513 is currently in phase 2, a single-blind, randomized, parallel-group, dose-ranging, single-dose study in India to study its safety and efficacy in adults with dengue fever (CTRI/2021/07/035290). AV-1, developed by AbViro LLC (NCT04273217), and Dengushield, developed by Serum Institute of India Pvt. Ltd. (NCT03883620) are other human monoclonal antibodies currently in phase 1, placebo-controlled, clinical trials with goals to determine the safety and pharmacokinetics in healthy adults. However, details on their preclinical development, target epitopes, and efficacy against DENV have not yet been published.

## Development of therapeutics targeting host

Many severe dengue symptoms are thought to result from a pathological host immune response [8]. Mast cells, in particular, have been identified as an important drug target for the treatment of severe dengue pathologies including vascular permeability, plasma leakage, and thrombocytopenia [89, 90]. Mast cells are granulated innate immune cells located along host-environment interfaces like epithelial and endothelial barriers such that they can quickly respond to invading pathogens [91]. During DENV infection in the skin, mast cells are activated in response to the virus and degranulate to release an extensive array of preformed mediators like lysosomal enzymes (β-hexosaminidase), biogenic amines, histamine and serotonin, mast cell-specific proteases–tryptase, chymase, and carboxypeptidase A3 and cytokines, such as TNF. In addition, mast cell activation leads to the de novo synthesis of lipid mediators—eicosanoids: leukotrienes and prostaglandins, and platelet activating factor (PAF), as well as other cytokines and chemokines, that subsequently aid recruitment of cytotoxic cells that help with viral clearance [92, 93]. In contrast to the localized protective response, pre-clinical studies with immunocompetent and immunocompromised mice showed that widespread mast cell activation during systemic DENV infection results in excessive release of vasoactive mast cell mediators including leukotrienes, PAF [94], and proteases tryptase and chymase [16, 18] that cause vascular leakage by disrupting the endothelium [17•]. Importantly, injection of tryptase at similar concentrations detected in the plasma of severe dengue patients was sufficient to induce a temperature drop indicative of shock in mice [17•]. Moreover, elevated tryptase levels correlated with increasing severity of DHF and DSS in humans [17•]. Tryptase inhibiting drug, nafamostat mesylate significantly reduced vascular leakage in DENV infected mice and was effective even after the delayed treatment [17•]. While tryptase function is specific to the vascular endothelium, serotonin released peripherally by mast cells during DENV infection was shown to induce thrombocytopenia [34•]. Activation of platelets by serotonin, sensed using 5HT2 receptors, triggered aggregation and destruction of platelets during DENV infection [34•]. Accordingly, treatment of mice using 5HT2 receptor antagonists, ketanserin and sarpogrelate reversed thrombocytopenia in DENV-infected mice [34•]. These studies highlighted that mast cell degranulation and mast cell products can be targeted in vivo with already clinically available drugs like mast cell stabilizers ketotifen and cromolyn [18], and inhibitors of mast cell products like nafamostat mesylate [17•] (for tryptase), montelukast (for leukotrienes) [18], ketanserin (for peripheral serotonin) [34•], and rupatadine (for PAF and histamine-1-receptor block) [94]. Ketotifen is currently in a phase 4 randomized clinical trial in Singapore that has concluded patient enrollment, where the primary endpoint of clinical fluid accumulation has been measured by MRI (NCT02673840). A preliminary randomized placebo-controlled clinical trial (SLCTR/2014/023) testing efficacy of rupatadine for acute dengue patients revealed improved platelet counts, reduction in liver transaminases suggesting reduced tissue inflammation, and reduction in volume of plural effusions on certain days of the study protocol in their post hoc analyses despite failing to meet its primary endpoint of reduction in fluid leakage (pleural effusion or ascites) [94]. Further studies are needed to evaluate the efficacy of rupatadine against dengue vasculopathy. A preliminary open labeled, randomized clinical trial in Pakistan provided some early evidence towards the efficacy of montelukast in reducing risk for DSS, as defined by narrow pulse pressure < 20 mmHg and hypotension for age [95]. Montelukast 10 mg was given once daily for 5 days. There was no change in hemoconcentration, or platelet counts although the overall DSS frequency reported in the cohort was very high (40%). The authors reported reduced incidence and relative risk of DSS in patients treated with montelukast, however the study lacks power analysis and a predefined significant statistical difference between the groups. A phase 2/3 multicentre, randomized, double-blind, placebo controlled, superiority trial (NCT04673422) is currently on-going in Thailand to study the effect of montelukast in preventing dengue with warning signs in dengue patients.

As metabolic disorders like obesity and diabetes are increasingly recognized risk factors for severe dengue [96•, 97•, 98, 99], metabolic drugs are being investigated as potential dengue therapeutics. For example, Metformin, is a well-established oral anti-hyperglycemic agent administered to diabetic patients. Its primary mechanism of action is to activate adenosine monophosphate (AMP)-activated protein kinase (AMPK), an important cellular energy sensor that is activated in conditions of low cellular energy levels to maintain homeostasis by upregulating lipid, protein, and glucose metabolism [100]. Interestingly, in vitro studies show that DENV impairs AMPK phosphorylation, which leads to downstream upregulation of HMG-CoA reductase activity, the rate-limiting step in cholesterol biosynthesis [101]. DENV proteins NS3 and NS4A colocalize in this resultant lipid-enriched environment which is shown to be conducive to enhanced DENV replication complex formation [101, 102]. Consistent with this mechanism of DENV replication, metformin showed a significant dose-dependent antiviral effect in DENV-infected cells in vitro [101]*.* Correspondingly, a retrospective study in confirmed dengue patients with diabetes showed that metformin use was associated with decreased risk of developing severe dengue, and further, there was a dose-dependent inverse relationship between metformin intake and dengue severity [103•]. However, it is unclear from this study whether the effects of metformin on diabetic control or on DENV replication may be linked to the use of this drug. Currently, an open-label, two-phase dose escalation trial (NCT04377451) of **m**etformin in **d**engue patients with **o**besity (MeDO) is on-going in Vietnam [104], which should clarify the therapeutic utility of metformin in dengue patients during acute disease.

## Other potential therapy–bioactive compounds

Many plant-based bioactive compounds have also been recognized to have anti-dengue activity in vitro and in vivo and have been summarized in a recent review [105]. Among these, Carica papaya leaf extract (CPLE) has been recognized for its platelet augmenting effect in both pediatric and adult dengue patients [107, 108]. Results from these randomized, placebo-controlled clinical trials showed that administration of CPLE thrice daily enhanced platelet recovery rate, with a statistically significant increase in platelet levels in the intervention group from day 3 of treatment. While the mechanism of action is not yet completely understood, a recent study indicates that CPLE increases the expression of thrombopoietin receptor, CD110, also known as the myeloproliferative leukemia protein (cMpl), on platelets and megakaryocytes which is important for megakaryocyte proliferation and platelet formation [109]. Micronutrients are significant immunomodulators [110] and thus are often prescribed as supplements in addition to standard supportive care for dengue patients [111, 112]. Clinical trials showed a reduction in liver derangements [113] and increased platelet recovery [114] upon vitamin E supplementation in addition to supportive therapy in dengue patients when compared to the control group. Other smaller RCTs with vitamin D [115] and zinc supplementation (TCTR20151110001) [116] showed reduced relative risk of DHF and decreased duration of hospital stay respectively. A retrospective observational study investigating the effect of vitamin C supplementation in dengue fever reported increased platelet recovery and reduced hospitalization duration in patients receiving vitamin C [117]. Currently, two phase-II clinical trials have been registered to study the efficacy of vitamin C (SLCTR/2017/028, Sri Lanka) and vitamin C and B1 combination (CTRI/2019/09/021244, India) in reducing morbidity in dengue patients.

## Vaccine development

Given the lack of specific dengue therapeutics and significant economic and public health burden, WHO considers dengue vaccine development an urgent priority [121]. Two main challenges prevail in dengue vaccine development. Firstly, the vaccine should confer effective and balanced protection against all four dengue serotypes. Tetravalent vaccine formulations developed with this aim are hindered by antigenic competition, wherein the immune response is skewed towards more immunodominant serotype-specific dengue antigens. Secondly, ADE poses a significant challenge in dengue vaccine development since vaccine-mediated sub-optimal protection to any of the dengue serotypes can confer a risk of severe dengue in a subsequent infection. Currently, there are at least seven dengue vaccines in various stages of development and clinical trials [122, 123•, 124–129]. We will discuss selected vaccine candidates that have progressed furthest in clinical development here.

CYD-TDV (**c**himeric **y**ellow fever virus–**D**ENV–**t**etravalent **d**engue **v**accine) aka Dengvaxia, developed by Sanofi Pasteur, was the first dengue vaccine to be licensed based on three clinical trials [130–132] and has been approved in certain countries, but is limited for use in individuals with prior dengue infection. It is a tetravalent live attenuated vaccine that uses the yellow fever YF17D vaccine strain as the backbone, substituting the prM and E proteins with the corresponding genes of the four wild-type dengue serotypes. It is administered subcutaneously as a 3-dose regimen scheduled 6 months apart in the population aged 9–45 years. In the pooled analysis of 25-month efficacy data from phase 3 trials in population aged 2–16 years (CYD14, CYD15 and CYD57) overall vaccine efficacy for symptomatic virologically confirmed dengue (VCD) was 60.3% (95% CI: 55.7–64.5). It has been proposed that the limited efficacy of the vaccine could result from the mismatched non-structural proteins from the YF17D backbone, potentially limiting the efficacy of the vaccine in inducing a protective T cell response against DENV [8]. Interestingly, for children, the vaccine efficacy was higher for those in older age groups with 65.6% (95% CI: 60.7–69.9) in those aged 9–16 years, than the younger age group with 44.6% (95% CI: 31.6–55.0) for those aged 2–8 years old [122]. For both age groups, specific vaccine efficacy for DENV1 and DENV2 was 40–50%, whereas it was 70–85% for DENV3 and DENV4. The vaccine seemed to be most efficacious against hospitalization and severe dengue with pooled vaccine efficacy across all age groups as 72.7% (95% CI: 62.3–80.3) and 79.1% (95% CI: 60.0–89.0) respectively, not stratified by serostatus [122]. Increased relative risk of hospitalization in children aged 2–9 years of 1.58% (95% CI: 0.83–3.02) versus 0.5% (95% CI: 0.29–0.86) in children aged 9–16 years was observed. Since 80% of the 9–16 years population were baseline seropositive, it was hypothesized that baseline serostatus of the patient could possibly influence dengue risk wherein the vaccine could potentially precipitate severe dengue in seronegative patients via ADE [133]. Indeed, vaccine efficacy was higher for seropositive individuals (70–80%) than for seronegative individuals (14.4–52.5%) for both age groups. A follow-up case-cohort study re-analyzing data from the three trials along with retrospective inference of serostatus of the individuals based on blood taken at 13 months post-vaccination, found that there was an increased risk of hospitalization and severe dengue in seronegative individuals [134]. The hazard ratio (HR) (vaccine vs. control) for hospitalization was 1.75 (95% CI: 1.14–2.70) and that for severe dengue was 2.87 (95% CI:1.09–7.61) in seronegative patients compared to corresponding HRs of 0.32 (95% CI: 0.23–0.45) and 0.31 (95% CI: 0.17–0.58) in seropositive patients. In accordance with this, the latest WHO Strategic Advisory Group of Experts on immunization recommends pre-vaccination screening as the preferred strategy for countries considering CYD-TDV vaccination as part of their dengue control program. With this strategy, only seropositive individuals with evidence of a laboratory or antibody test confirmed past dengue infection would be vaccinated. If pre-vaccination screening is not feasible, vaccination can still be considered if the recent seroprevalence rate in the area has been documented to be at least 80% by 9 years of age [135].

QDENGA® or TAK-003 by Takeda Pharmaceuticals is another live-attenuated tetravalent dengue vaccine that has been recently licensed for use in Indonesia for individuals aged 6 to 45 [136], and has received marketing authorization in the European Union for individuals aged 4 and above [137], regardless of their prior dengue exposure status. It is currently undergoing regulatory review in other dengue-endemic countries in Asia and Latin America [138, 139]. Using attenuated DENV2 PDK-53 virus as backbone, the other chimeric dengue virus were engineered by substituting the prM and E genes of DENV2 for that of wild-type DENV1 16007, DENV3 16562, or DENV4 1036 virus [140]. It is administered subcutaneously as a 0.5 mL dose in a two dose regimen scheduled 3 months apart [136, 137]. The approval is based on the Tetravalent Immunization against Dengue Efficacy Study (TIDES) trial which included more than 28,000 participants. The primary endpoint of the study was the overall vaccine efficacy against VCD in the first 11 months and was found to be 80·2% (95% CI: 73·3–85·3) [141]. However, subsequent follow-up studies at 18-, 24- and 36-months vaccination showed waning of vaccine efficacy over time with cumulative vaccine efficacy against VCD at 62% (95% CI: 56.6–66.7) [123•, 124–143]. Nonetheless, vaccine efficacy against hospitalized VCD was more robust and maintained from 90.4% (95% CI: 82.6–94.7) at 18 months to 83.6% (95% CI: 76.8–88.4) at 3 years after the second dose. Importantly, the year 3 cumulative data indicated no age effect and showed comparable overall vaccine efficacy against VCD (65% vs 54.3%) and hospitalized VCD (86% vs 77.1%) for seropositive and seronegative individuals. When cumulative vaccine efficacy was analyzed for specific serotypes, similar vaccine efficacy regardless of baseline serostatus was observed for DENV-1 (seropositive: 56.2% (95% CI: 43.7–66.0) vs seronegative: 43.5% (95% CI: 21.5–59.3)) and DENV2 (seropositive: 83.4% (95% CI: 76.4–88.3) vs seronegative: 91.9% (95% CI: 83.6–96.0)). However, while vaccine efficacy was 52.3% (95% CI: 36.6–64.2) for DENV-3 and 60.7% (95% CI: 16.0–81.6) for DENV-4 in seropositive individuals, no efficacy was observed for DENV-3 (− 23.4% (95% CI: − 125.3–32.4)) and efficacy against DENV-4 was inconclusive in seronegative patients [123•]. Notably seronegative patients showed higher hospitalization rate (0.2%) compared to placebo (< 0.1%) for DENV-3 infection [123•], raising concerns that serostatus might also affect safety outcomes with this vaccine, similarly to Dengvaxia [144]. While this relative risk was inconclusive and was attributed by the authors to the small number of DENV-3 cases and differences in clinical practice geographically, further analyses are warranted to ensure no increased hazard risk due to ADE in seronegative patients. Moreover, the gradual waning efficacy needs to be addressed. A new clinical trial (NCT03999996) to assess the impact of a booster dose 15- and 24-months post 1^st^ dose of vaccine is currently on-going. In addition, the presence of non-structural (NS) proteins from the DENV-2 backbone in Qdenga was shown to elicit NS-proteins specific T-cell mediated immunity in samples from phase 2 trial [145], however, it is notable that the protection offered by this vaccine is highest against DENV2, the strain from which the virus backbone originates. This might point to the importance of ensuring that future DENV vaccine development prioritizes generating tetravalent immunity with respect to both antibodies and T cell epitopes.

TV003/TV005, developed by National Institute of Allergy and Infectious Diseases (NIAID), is a live attenuated virus vaccine in clinical testing. TV003/TV005 was developed by introducing 30 nucleotide deletions in the 3’ untranslated region and additional mutations in non-structural proteins to engineer live attenuated dengue virus—rDEN1Δ30, rDEN3Δ30/31 and rDEN4Δ30. Attenuated DENV-2 virus was engineered as a chimeric virus by substituting the prM and E proteins in rDEN4Δ30 with that of DENV-2 [146]. TV003 (10^3^ PFU) and TV005 (10^4^ PFU) differ in the dosage of rDEN2/4Δ30 component. Two randomized placebo controlled phase I trials evaluating safety and immunogenicity of TV003 and TV005 found a low grade rash as the most frequent adverse reaction [147, 148]. Importantly, pooled analysis from both the trials showed between 64%-100% seroconversion following a single dose of TV003 [149]. Compared to placebo, TV003 induced serotype specific neutralizing antibodies and showed protection against viremia, rash, and neutropenia when challenged with an attenuated DENV2 strain, rDEN2Δ30, 6-months post vaccination [150]. TV003 has since been licensed by the Butantan Institute in Brazil and manufactured as a lyophilized TDV named Butantan-DV. A double blind, randomized, placebo-controlled phase II trial showed seroconversion for all four dengue serotypes 91-days post single dose of Butantan-DV in both seronegative (76%-92%) and seropositive (77–82%) individuals with rash as the main adverse reaction [124]. Seroconversion was defined by PRNT50 cutoff titers (≥ 1/10) for seronegative individuals and as a four-fold or higher increase in pre-existing neutralizing antibody titer after immunization for the seropositive individuals. Butantan-DV elicited neutralizing antibodies against all four DENV serotypes with 75% of the participants developing tetravalent neutralizing humoral response after a single vaccine dose. Notably, the neutralizing antibody geometric mean titer was significantly higher in DENV-exposed individuals than DENV-naive participants for DENV-1, DENV-2, and DENV-3, but not for DENV-4 (*p* = 0·077). Presence of non-structural proteins of three different serotypes in Butantan-DV also initiated cellular immune response as evidenced by antigen specific CD8 + T cell responses when stimulated ex vivo with DENV-derived peptide pool in 94% of the vaccinated individuals as compared to 13% in placebo 91 days post first dose of vaccination [124]. This vaccine is currently in phase III clinical trial in Brazil with 16,944 participants divided into three age groups (18–59 years, 7–17 years, and 2–6 years).

## Conclusion

The current standard of care for dengue remains supportive care. Timely fluid management has shown to be effective in lowering deaths due to severe dengue. However, due to the late onset of warning signs, it can be difficult to manage severe dengue solely guided by symptomatic interventions and is often too late too little for the interventions to be effective. Therefore, there is a dire need for effective drugs that can reduce dengue viral infection and reduce the likelihood of developing severe disease. Moreover, an integrated approach utilizing both efficacious vaccines and drugs will be required to combat dengue globally. As highlighted in this review, there are new and promising drugs and vaccines for the control of dengue at various stages of clinical development.
